# High Rates of Unplanned Care Interruption: Implications for Program Response

**DOI:** 10.4172/2375-4273.1000146

**Published:** 2015

**Authors:** Bolanle Banigbe, Abdulkabir B. Adegoke, Kenneth A. Freedberg, Prosper Okonkwo, Aimalohi A. Ahonkhai

**Affiliations:** 1 AIDS Prevention Initiative in Nigeria (APIN), Abuja, Nigeria; 2 Medical Practice Evaluation Center, Massachusetts General Hospital, Boston, Massachusetts, USA; 3 Division of Infectious Disease, Massachusetts General Hospital, Boston, Massachusetts, USA; 4 Harvard Medical School, Boston, Massachusetts, USA; 5 Division of General Internal Medicine, Massachusetts General Hospital, Boston, Massachusetts, USA; 6 Harvard University Center for AIDS Research (CFAR), Boston, Massachusetts, USA; 7 Department of Health Policy and Management, Harvard T.H. Chan School of Public Health, Boston, Massachusetts, USA

## Abstract

Unplanned care interruption (UCI) is an important challenge for HIV programs in resource- limited settings (RLS). More than 1 in 3 patients will interrupt care after starting antiretroviral therapy (ART), predisposing them to poor clinical outcomes. As HIV programs in RLS adopt the new World Health Organization (WHO) treatment guidelines recommending ART for all patients, the volume of patients requiring ART, and the number of patients with UCIs will increase. In addition, reduced donor funding may drive changes at the local level that create additional barriers to care. Policy makers and program managers therefore need to adopt innovative care models to enhance retention in care. The integration of patient-centered chronic care models into HIV care delivery may provide a template for addressing these challenges while serving as a model of care for other chronic diseases. Fortunately, many President’s Emergency Fund for AIDS Relief (PEPFAR) supported HIV clinics have already incorporated some important elements of chronic care models. However, strategic efforts are needed to strengthen and develop them into comprehensive approaches to HIV care in this new era of care delivery.

Effective scale up of antiretroviral therapy (ART) has resulted in improved quality of life, and reduced morbidity and mortality on a global scale [1]. Despite these gains, long term retention in care remains a challenge for many HIV treatment programs, especially in resource-limited settings (RLS) [2]. There are many potential measures of retention, and much of the retention literature has focused on patients who are lost to follow-up, despite its limitations [2]. Loss to follow-up (LTFU), as a measure, may be conflated with other poorly ascertained outcomes including undocumented transfers of care, death, or transient care interruptions [3].

Our group has recently published on unplanned care interruption (UCI) as a measure of retention, a less heterogeneous outcome that can be assessed at the program level without requiring resources to track patients who do not return to care [3]. Our recent analysis was based in Nigeria, home to the second largest number of people in the world living with HIV after South Africa [1]. In our cohort, 35% of patients had UCI, and rates were highest in the first year after starting ART [3]. This study supports our experience in the field – that patients are likely to experience many episodes of UCI over the course of their care. Not surprisingly, these interruptions are often associated with medication lapses, resulting in CD4 decline and development of viremia [4]. Despite this common finding in programs, the alarming frequency of unplanned interruptions from care is just beginning to find a greater voice in the literature [4].

In our analysis of one cohort in Northern Nigeria, we found that having a higher baseline CD4 count (>350 cells/μL) was associated with a 3-fold increased risk of UCI in the first year on ART. This may reflect a less robust commitment to care among patients who generally do not feel “sick” [2,3]. Indeed patients who do not feel ill may be less willing to invest in travelling to clinic (because of cost of transportation, potential lost wages, etc.) and taking medications (with potential toxicities and side effects) than patients who feel more ill [2].

Over the past 10 years, the World Health Organization (WHO) has steadily increased the threshold CD4 count for ART initiation in response to evidence supporting improved morbidity and mortality for patients who start therapy earlier [5,6]. The recently published Strategic Timing of Antiretroviral Treatment (START) trial established continued benefit of early ART for patients with the highest CD4 counts (>500cells/μL), thus motivating the WHO to recommend ART for all individuals infected with HIV regardless of CD4 count [5,6]. The WHO’s new “treat all” strategy will make an additional 22.1 million people eligible for ART globally, approximately 1 in 8 of those will be in Nigeria [6]. While Nigeria and other RLS have not yet adopted WHO’s most recent guidelines, without enacting a strategic response to these recommendations, the potential for unintended consequences are great. First, the larger volume of patients requiring ART will increase the standard caseload of health care workers who are already overburdened. In addition, many of the patients starting ART will do so at higher CD4 counts, and thus be at greater risk for care interruption, with subsequent immunologic decline, and virologic failure [3]. Some will develop drug resistance and require more costly 2nd and 3rd. line ART regimens [4]. Fragile, stretched health systems may be further taxed to increase patient outreach to improve retention in care, and to address the clinical consequences of poor disease control in this context.

Despite these challenges, with the clear benefit of early ART, program managers and local governments in RLS must think of novel strategies to ensure retention in care that will remain effective in a climate of reduced donor funding. Notably, the success of the global AIDS response, especially in RLS, has relied heavily on financial resources from multilateral institutions and donor as well as host-country governments. The President’s Emergency Fund for AIDS Relief (PEPFAR); the U.S. Government initiative to initiative to combat global HIV/AIDS, TB, and malaria in 15 hard hit “focus countries has contributed over $50 billion to this effort [7]. This support has made HIV care and treatment services largely free to patients in these countries. Since 2010, PEPFAR has worked with recipient countries to increase their commitments to HIV programs; the Nigerian government committed to increase its contribution to the national HIV/AIDS response from 7% in 2008 to 50% by 2015 [8]. Consequently PEPFAR’s funding to Nigeria has decreased by $44 million [9]. As future funding remains uncertain, the gap between available resources and anticipated needs may widen.

In an effort to bridge this funding gap, many programs have instituted clinic-based user fees to maintain program operations. Point-of care fees are typical in Nigeria, in fact, out-of- pocket healthcare expenditures make up an extraordinary 96% of total healthcare costs overall [10]. However, until recently, HIV care has been a remarkable exception to this trend. Still, more than 80% of Nigerians live on less than 2 US dollars per day; those who are HIV- infected may be forced to negotiate between basic subsistence and HIV-specific healthcare needs [10]. Studies from low and middle-income countries in the early ART scale-up era (in which fees were commonly charged for ART) reported that clinics with user fees had a 4 to

5-fold increased risk of attrition and death [11]. Another consequence of reduced donor funding has been the imposition of strict salary caps for clinic staff, which has also resulted in staff attrition at the HIV clinics. This has especially affected non-medical staff including those responsible for patient outreach for patients who are lost to follow-up.

With the advent and widespread use of potent highly active ART, HIV has transformed into a very treatable, chronic disease [1]. In this context, successful paradigms developed for HIV care in RLS can be utilized as models for other chronic diseases. While few comprehensive frameworks exist to evaluate HIV care delivery systems in RLS, models adopting a patient- centered, chronic care approach may provide an excellent starting point. One such example is the patient-centered medical home (PCMH). The PCMH promotes comprehensive care coordinated across all the elements of the health care system, and espouses accessible, continuous, comprehensive, family-centered, coordinated, and culturally competent care. Through 6 core pillars [12]. Early evidence suggests that the PCMH may improve quality and reduce cost of care for certain chronic medical conditions in the United States [12]. While not yet well studied in RLS, a recent analysis by our group found that our large treatment network performed well according to this standard, and the PCMH scoring tool helped identify system-wide targets for improvement, and opportunities to identify and share best practices across the network [13].

Another framework that has received increasing attention is the chronic care model (CCM). The CCM has been used extensively in the management of non-communicable diseases (NCDs) in high income countries and HIV care settings [14]. Like the PCMH, the CCM advocates coordinated care at the clinic, community and individual level, and incorporates 6 care elements: 1) clinical information systems optimized to facilitate long-term disease management, 2) delivery systems designed to be efficient and proactive, 3) decision support to help providers exercise sound, evidence-based clinical judgment, and 4) self- management support to help patients negotiate the daily challenges and choices involved in providing good self-care. These components are embedded in two additional CCM components: 5) supportive health system leadership and 6) complementary community resources.

As PEPFAR and other global partners for HIV care transition from an emergency response agenda to one of sustainable programs, building on billions of dollars’ worth of health sector investments, clinics at the frontline of HIV care are compelled to adapt their service delivery models to ensure longevity. While the experience with efficiently managing chronic diseases is limited in Nigeria as in many other RLS, HIV treatment programs may provide an exception to this. The scale-up of HIV treatment services has organically included some elements of comprehensive chronic care models, including a focus on robust medical record systems, and electronic medical records to facilitate longitudinal patient data that allows providers to track upcoming appointments, identify missed visits, and document important

Outcomes [12,14]. This constitutes a major step in the direction of clinical information systems that facilitate long-term disease management. Another unique element of HIV treatment programs that distinguishes them from the care of other chronic diseases in RLS is a multidisciplinary approach to care incorporating medication readiness programs, counseling and outreach services (including home-based care teams, peer support), in addition to local pharmacy and clinical services [12]. Health education in the promotion of self-management and decision support is another important element of chronic care that is fairly unique to HIV care programs in RLS [14]. HIV-specific outcomes hinge on access and adherence to antiretroviral medications, and the importance of this is incorporated into clinic and peer-based education and adherence activities in most clinics. These care elements form a foundation for comprehensive self-management support that can empower patients to make informed choices and promote behavior change to improve outcomes. Some important chronic care elements, including patient and family- centered care calling for individualized care plans, are, however, endorsed by both the CCM and PCMH but lacking in the Nigerian HIV programs [12,14]. Integration of patient-centered care plans will require a change in the provider-patient power dynamic that still makes it very difficult for patients to participate in decision-making about their own care in many RLS. In conclusion, UCI is an important reality of HIV care. UCI may become more prevalent as policy changes are enacted that increase the population of early stage patients eligible for ART, while also increasing the out-of-pocket expenses required for HIV care. Creative solutions that prioritize patients’ health goals while addressing chronic health needs are required at the clinic, program and government levels. Successful integration of a chronic care model for HIV into the Nigerian health care system could help deliver affordable high- quality healthcare, and serve as a model for the management of other chronic diseases. PEPFAR’s recent decision to embrace an implementation science framework in its implementation is as an important opportunity to test and adapt models for chronic disease management in RLS and to disseminate widely, work that is ongoing in PEPFAR countries [15].
